# Comparison of the Snare Loop Technique and the Hungaroring Reinforcement for Physician-Modified Endograft Fenestrations—An In Vitro Study

**DOI:** 10.3390/jcdd11050134

**Published:** 2024-04-25

**Authors:** Artúr Hüttl, Tin Dat Nguyen, Sarolta Borzsák, András Süvegh, András Szentiványi, István Szilvácsku, Dóra Kovács, János Dobránszky, Péter Sótonyi, Csaba Csobay-Novák

**Affiliations:** 1 Department of Interventional Radiology, Heart and Vascular Center, Semmelweis University, Városmajor u. 68., 1122 Budapest, Hungary; huttl.artur@semmelweis.hu (A.H.); nguyen.tin.dat@semmelweis.hu (T.D.N.); borzsak.sarolta@semmelweis.hu (S.B.); suvegh.andras@stud.semmelweis.hu (A.S.); szentivanyi.andras@stud.semmelweis.hu (A.S.); 2 Semmelweis Aortic Center, Semmelweis University, Városmajor u. 68., 1122 Budapest, Hungary; sotonyi.peter@semmelweis.hu; 3 Department of Materials Science and Engineering, Budapest University of Technology and Economics, Műegyetem rkp. 3-9, 1111 Budapest, Hungary; szilvacskuisti@gmail.com (I.S.); karoly.dora@gmail.com (D.K.); 4 ELKH-BME Research Group for Composite Science and Technology, Műegyetem rkp. 3-9, 1111 Budapest, Hungary; dobranszky.janos@eik.bme.hu; 5 Department of Vascular and Endovascular Surgery, Heart and Vascular Center, Semmelweis University, Városmajor u. 68., 1122 Budapest, Hungary

**Keywords:** endovascular aortic repair, physician-modified endograft, fenestration, reinforcement

## Abstract

Background: We conducted an in vitro comparison of the snare loop reinforcement against a closed-loop reinforcement (Hungaroring) for physician-modified endograft (PMEG) fenestrations regarding preparation time and stability during flaring balloon dilatation. Materials and methods: The time to complete a PMEG fenestration with reinforcement was measured and compared between the Hungaroring and snare loop groups. The number of stitches was counted. Each fenestration was dilated using a 10 mm high-pressure, non-compliant balloon up to 21 atm in pressure, and fluoroscopic images were taken. The presence of indentation on the oversized balloon at the level of the reinforcement was evaluated at each fenestration. Results: Five fenestrations were created in each group (n = 5) for a total of ten pieces. The completion time in the snare loop group was 1070 s (IQR:1010–1090) compared to 760 s (IQR:685–784) in the Hungaroring group (*p* = 0.008). Faster completion time was achieved by faster stitching (23.2 s/stitch (IQR 22.8–27.3) for the snare loop group and 17.3 s/stitch (IQR 17.3–20.1) for the Hungaroring group (*p* = 0.016). None of the fluoroscopic images of the snare loop reinforcement showed an indentation on the balloon during the overexpansion; on the contrary, the Hungaroring showed indentation in every case, even at 21 atm. Conclusion: Fenestrations reinforced with Hungaroring can be completed significantly faster. Furthermore, the Hungaroring resists over-dilation even at high pressures, while snare loop reinforcements dilate at nominal pressure.

## 1. Introduction

Nowadays, thanks to technical evolution, endovascular treatment is feasible for thoracoabdominal and complex abdominal aortic aneurysms. To achieve the patency of the abdominal visceral branches, fenestrated or branched endovascular aortic repair (F/BEVAR) is needed. In some patients, anatomical specificities disable the use of off-the-shelf devices but require the use of custom-made devices (CMDs) tailored to the specific anatomy. However, the production time of such CMDs can take an average of 15 weeks [1]. Thus, to manage complex aortic pathologies requiring urgent or emergent endovascular treatment, off-the-shelf devices [2], parallel grafts [3], in situ fenestrations [4,5] and physician-modified endografts (PMEGs) [6,7] can be used [8]. For contemporary endovascular interventions involving three or four branch vessels, CMDs have become the first choice. This preference stems from the increased likelihood of developing gutter endoleaks when using parallel-guided grafts in such cases. The use of custom-made endovascular devices tailored to the anatomy of individual patients contributes significantly to the overall expenditure. With the increasing frequency of such endovascular procedures, the growing financial burden on healthcare systems necessitates the exploration of alternative approaches. The widespread use of PMEG could offer a potential solution to address both cost implications and clinical outcomes. Clinical studies show that there is no difference between the results of aneurysm reconstruction with CMD or PMEG [9].

The term PMEG was coined by Starnes et al. as a summary of all the modifications of a readily available endograft that can be performed before implantation while maintaining the sterility of the device [10]. A key step in the preparation process of these devices is the formation of the side branch fenestrations and their reinforcement with a marker. The most widely accepted technique is to create the fenestration with cautery and reinforce the fenestration by either a snare loop or a guidewire tip stitched around the fenestration with braided polyester sutures [7,11]. These two techniques result in an open-ring reinforcement around the fenestration, which was shown to carry an inherent risk of distortion during flaring associated with the slippage of the unconnected ends [12]. Although the reported incidence of late-type IIIc endoleaks after PMEG repair is currently low, this design might be associated with failure in the long term [12,13]. 

To overcome the disadvantages of such open-ring reinforcements, a new tool was developed to achieve a design that is resistant to over-dilatation during the flaring maneuver, similar to that of fenestrations on CMDs. The Hungaroring itself is a closed-loop side branch fenestration reinforcement that can be used during PMEG preparation to mark and stabilize the cut-out fenestrations on the endograft. It is made of commercially available raw materials and can be prepared by anybody with simple tools, resulting in a device that can be created in advance, sterilized, and stored for later use in urgent situations [14]. 

The purpose of this study was to compare the snare loop reinforcement against the Hungaroring in terms of preparation time and stability during balloon overexpansion in an in vitro model of the flaring maneuver. Our primary aim was to demonstrate that PMEG fenestrations reinforced with the Hungaroring can be safely flared with an oversized balloon that can be expanded, even up to the rated burst pressure (RBP), without the fear of distorting the fenestration.

## 2. Materials and Methods

In our in vitro study, we compared the conventional snare loop reinforcement with the Hungaroring (closed loop) reinforcement. 

For this purpose, we first prepared Hungaroring rings. To make a Hungaroring with a diameter of 8 mm, both ends of a 100 mm long ultrathin nitinol wire (Fort Wayne Metals, Fort Wayne, IN, USA) were connected into a ring with a short tantalum tube as a crimp ferrule (Heeger Materials, Denver, CO, USA), using a crimping process as we previously reported [14]. Three more pieces of tantalum ferrule were positioned over the wire as X-ray markers around the fenestration. The large circle was folded in half twice, resulting in an 8 mm ring with four wires running. The ring was stabilized with knots to prevent it from jumping back to its larger configuration.

The first step in our in vitro test series was to measure the time to complete the complete suture of fenestration with the Hungaroring and a snare loop, respectively. The fenestrations and their reinforcements were always made by the same two operators. Before the test, both methods were practiced thoroughly until the fenestration preparation and the sewing were performed at a steady, brisk pace. Thereafter, the trial measurements showed no large variation in the time taken to complete the confirmations using the same method.

This was followed by live tests. In both groups, we created two sets of five 8 mm holes using a cautery device on a thoracic prosthesis (Valiant, Medtronic, Dublin, Ireland) stent graft. The prepared snare loops and the Hungarorings were sewn around the fenestrations using 5–0 braided polyester (Ethibond; Ethicon Inc., Raritan, NJ, USA) sutures. For each of the ten fenestrations, we measured the total time taken to complete the fenestration from the time the hole was created to the time the last stitch was knotted. 

The number of stitches was counted on high-magnification images, and the average time for one suture was calculated. 

In a second step, all the reinforced fenestrations were dilated using a 10 mm, non-compliant high-pressure balloon with a nominal pressure (NP) of 6 atm and RBP of 14 atm (Mustang; Boston Scientific, Marlborough, MA, USA) sequentially at 10 atm, 14 atm and finally at 21 atm. Fluoroscopic images were taken during dilatation using fixed-mount angiography equipment (Artis Zee, Siemens Healthineers, Erlangen, Germany). A plane perpendicular to the axis of the balloon was used in order to visualize the indentation at the level of the reinforcement ring, which is the angiographic evidence of proper flaring in real-life FEVAR. High-resolution still images were acquired in each fenestration at 10 atm and 14 atm (RBP). 

Statistical analysis was performed using SPSS Statistics for Windows (Version 25.0.; IBM Corp., Armonk, NY, USA). Continuous data were expressed as the median and interquartile range (IQR) and compared using the Mann–Whitney U test. A two-sided *p*-value ≤ 0.05 was considered statistically significant.

## 3. Results

All in all, ten fenestrations were generated, with five using the snare loop (S1–S5; see Table 1) and an additional five employing the Hungaroring reinforcement (H1–H5; see Table 1). In all cases, the fenestrations started were successfully completed, so the technical success rate was 100%.

The median completion time in the snare loop group was 1070 s (IQR:1010–1090), compared to 760 s (IQR:685–784) in the Hungaroring group. The preparation time was significantly lower where Hungaroring was used (*p* = 0.008), but there was no statistically significant difference in the number of stitches (*p* = 0.222). The average time for one stitch was significantly lower in the Hungaroring group (*p* = 0.016; see Table 1).

**Figure 1:**
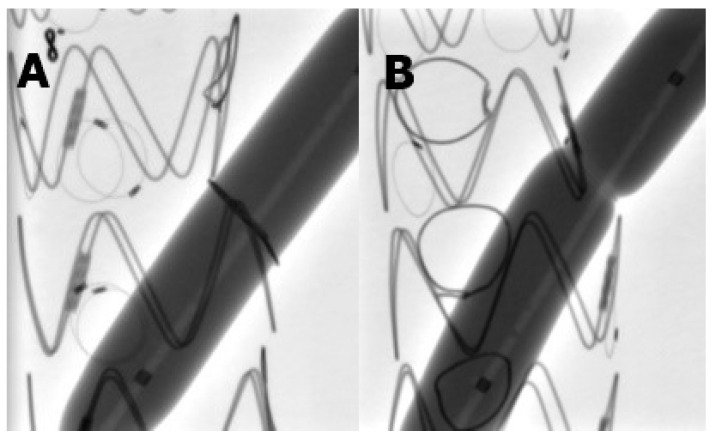
(**A**) No indentation even below 8 atm (**B**) Hungaroring resisted over-expansion even at 21 atm.

**Figure 2:**
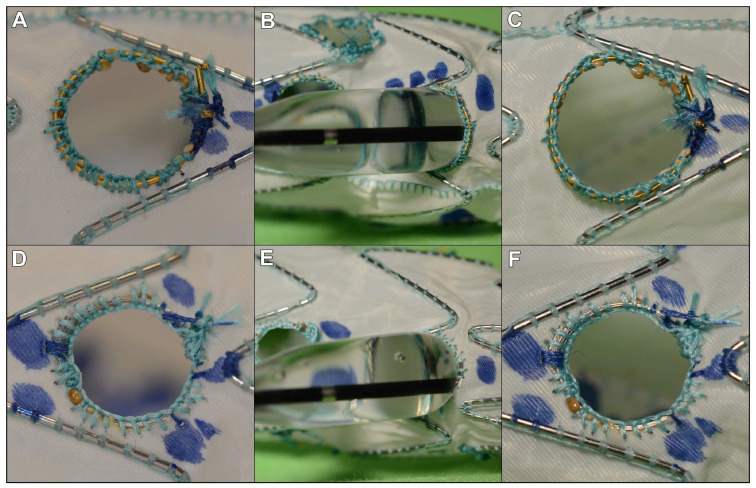
Macro images of the fenestration reinforcement both pre- and post-dilation. (**A**) Snare loop reinforcement pre-dilation; (**B**) flaring maneuver with no indentation; (**C**) snare loop reinforcement post-dilation; (**D**) Hungaroring pre-dilation; (**E**) flaring maneuver with visible indentation; and (**F**) post-dilation.

During balloon dilatation tests, all fenestrations reinforced with a snare loop demonstrated an effacement of the indentation already at pressures below the nominal pressure at 6 atm (Figure 1A). On the contrary, the Hungaroring resisted over-expansion even at 21 atm, which is way above RBP in every case (Figure 1B). High-resolution and high-magnification macro images show that Hungaroring resisted the expansion without any significant and visible distortion (Figure 2). 

The balloon burst at 24 atm without any effect on the Hungaroring reinforcement ring.

## 4. Discussion

The aim of this study was to demonstrate that the closed-ring reinforcement of a PMEG fenestration can be flared safely with an oversized balloon at extreme pressures without the risk of distorting the ring. We also compared the snare loop reinforcement with Hungaroring in terms of preparation time. Our results show that it is significantly faster to reinforce a fenestration with Hungaroring than with a snare loop. Hungaroring was already used in vivo, where we used this reinforcement technique to treat an internal iliac aneurysm with a fenestrated iliac limb, as previously reported [14]. 

Complex aortic interventions using the PMEG technique are still performed only in a limited number and in a few centers. The first PMEG series in a cohort of 47 patients was reported by Starnes et al. in 2012 [10]. They created the fenestrations using an ophthalmic Bovie cautery device. They then sutured the end of a gold, 15 mm Amplatz Gooseneck Snare around the opening using 4–0 Prolene sutures [10]. Oderich et al. reinforced the fenestrations with gold nitinol wire from a snare loop. The gold wire was sutured using a 5–0 Prolene suture [15]. 

In addition, in situ fenestration is increasingly performed, most commonly with a laser or radiofrequency device, following the implantation of a stent graft. In this case, no reinforcement is used around the orifice. These two previously described fenestration techniques were compared by Crawford et al. in their article published in 2016 [16]. Based on their results, short-term results showed that type IIIc endoleaks did not form more frequently in fenestrations without reinforcement. Eadie et al. found that the diameter of these fenestrations increased during a cyclic fatigue test following in situ fenestration [17]. Although current data show a low and late complication rate, concerns remain regarding the durability of the assembly in the long term [12].

In a recent paper, Canonge et al. compared the mechanical properties of various fenestration techniques currently used in the literature under in vitro conditions [12]. In their comprehensive and thorough study, they found that fenestration reinforcement improved fenestration stability and resistance. However, even double nitinol loops expanded during balloon dilatation and showed distortion in the cyclical fatigue test. They also concluded that the radial force applied to the bridging stent depends on the deformability of the fenestration. A stiffer fenestration means a higher radial force, and the bridging stent is expected to be more securely positioned. This suggests that the Hungaroring, which withstood even 24 atm of over-expansion, may provide good long-term results.

Jayet et al. tested two commercially available fenestrated stent grafts under in vitro conditions [18]. These custom-made devices have fenestrations that are reinforced with nitinol rings, similar to the Hungaroring. They found that the diameter of the fenestrations increased during the cyclic fatigue test, but there was a significant difference between the two stent graft systems tested. These notable results suggest that the material of the stent graft, the type of reinforcement as well as the anchorage of the reinforcement may affect the long-term stability of the fenestrations [18].

Another interesting and clinically useful innovation may be hydrogel-enhanced refenestration, as recently reported by Azuma et al. [19]. The reinforcement of restenestrations may thus allow the treatment of not only juxtarenal but also complex thoracoabdominal aneurysms in the future. However, the question is their long-term efficacy and stability so that they can be used even in younger patients without the fear of developing endoleaks. The in vitro articles cited above also seek to answer this question in terms of which forces act in the long term and what degradation these cause in the implanted devices. We believe and have demonstrated in our experiments that the Hungaroring we use is more resistant to mechanical stresses than the snare loop reinforcement currently used.

## 5. Conclusions

In our in vitro study, we demonstrated that fenestrations reinforced with a Hungaroring can be safely over-dilated even at extreme pressures without the risk of distorting the ring. Contrary to that, snare loop reinforcement held no strength to resist over-dilatation when already at low pressures. The use of a closed-ring reinforcement technique might increase side branch stability in the long term, which may result in a decrease in type IIIc endoleaks over time. 

Furthermore, Hungaroring reinforcement was completed significantly faster compared to snare loop reinforcement, regardless of the total number of stitches applied.

## Figures and Tables

**Table 1:** Measurement results in the snare loop group and Hungaroring group (the median value and the interquartile range are given).

	Snare Loop (n = 5)	Hungaroring (n = 5)	*p* Value
Completion time (s)	1070 (1010–1090)	760 (685–784)	0.008
No. of sutures	47 (39–48)	40 (38.5–42.5)	0.22
Average time/stitch (s)	23.2 (21.65–27.9)	17.3 (17.2–20.35)	0.016

## Data Availability

The data are contained within the article.
